# The Spatial Distribution of Ankle Muscles Activity Discriminates Aged from Young Subjects during Standing

**DOI:** 10.3389/fnhum.2017.00190

**Published:** 2017-04-19

**Authors:** Fabio V. dos Anjos, Talita P. Pinto, Marco Gazzoni, Taian M. Vieira

**Affiliations:** Laboratorio di Ingegneria del Sistema Neuromuscolare, Dipartimento di Elettronica e Telecomunicazioni, Politecnico di TorinoTorino, Italy

**Keywords:** postural control, standing, aging, electromyography, muscle activity

## Abstract

During standing, age-related differences in the activation of ankle muscles have been reported from surface electromyograms (EMGs) sampled locally. Given though activity seems to distribute unevenly within ankle muscles, the local sampling of surface EMGs may provide a biased view on how often and how much elderly and young individuals activate these muscles during standing. This study aimed therefore at sampling EMGs from multiple regions of individual ankle muscles to evaluate whether the distribution of muscle activity differs between aged and young subjects during standing. Thirteen young and eleven aged, healthy subjects were tested. Surface EMGs were sampled at multiple skin locations from tibialis anterior, soleus and medial and lateral gastrocnemius muscles while subjects stood at ease. The root mean square amplitude of EMGs was considered to estimate the duration, the degree of activity and the size of the region where muscle activity was detected. Our main findings revealed the medial gastrocnemius was active for longer periods in aged (interquartile interval; 74.1–98.2%) than young (44.9–81.9%) individuals (*P* = 0.02). Similarly, while tibialis anterior was rarely active in young (0.7–4.4%), in elderly subjects (2.6–82.5%) it was often recruited (*P* = 0.01). Moreover, EMGs with relatively higher amplitude were detected over a significantly wider proximo-distal region of medial gastrocnemius in aged (29.4–45.6%) than young (20.1–31.3%) subjects (*P* = 0.04). These results indicate the duration and the size of active muscle volume, as quantified from the spatial distribution of surface EMGs, may discriminate aged from young individuals during standing; elderlies seem to rely more heavily on the active loading of ankle muscles to control their standing posture than young individuals. Most importantly, current results suggest different conclusions on the active control of standing posture may be drawn depending on the skin location from where EMGs are collected, in particular for the medial gastrocnemius.

## Introduction

Different mechanisms have been suggested to account for the control of human, standing posture. Of recent interest is the notion that bodily sways are arrested by timely, triggered bursts of calf muscles' activity (Loram et al., 2005; Vieira et al., 2012; Héroux et al., 2014). The physiological mechanism underpinning timed activation in standing is controversial; some suggest there is an internal clock triggering calf muscles' activation (Loram et al., 2009) while others believe activation is event-triggered (Bottaro et al., 2008). In spite of the potential sources accounting for such intermittent, postural activation, alternating periods of muscle activation with silencing seems advantageous. Periodically silencing postural muscles during standing may: (i) allow the nervous system to sense joint angles without interference from muscle contraction (Loram et al., 2011); (ii) compensate for intrinsic, feedback delays (Cabrera and Milton, 2002); (iii) reduce metabolic costs (Bottaro et al., 2008); (iv) not increase postural instability (Cabrera and Milton, 2002). In this view, the assessment of muscle activation may therefore reveal pivotal features of the control of standing posture, especially e.g., in persons with balance impairments.

Surface EMGs revealed, indeed, key differences in the activation of postural muscles with aging. From bipolar surface EMGs collected from the plantar flexor muscles, for example, previous studies reported that elderlies tend to stand with a more continuous and higher degree of activation than young individuals (Laughton et al., 2003; Nagai et al., 2011; Baudry et al., 2012). Moreover, while tibialis anterior is typically silent in young subjects during standing (Di Giulio et al., 2009; Masani et al., 2013), in aged individuals it is often recruited (Laughton et al., 2003). From the point of view of muscle activation, it seems therefore standing becomes progressively more demanding with aging. A crucial question arising from previous studies is whether the greater, prolonged activation observed locally (i.e., with bipolar EMGs) in plantar and dorsal flexors of aged subjects reflects the activation of the muscle as a whole.

The spatial distribution of surface EMGs over the whole muscle rather than the local sampling of surface EMGs with bipolar electrodes seems to more likely provide a genuine indication on the duration and degree of muscle activity. EMGs with different amplitudes have indeed been observed when sampled from different regions of a single muscle (Brown et al., 2007; Farina et al., 2008), suggesting activity does not distribute uniformly within the muscle volume. This uneven distribution of activity has been often observed for the calf muscles and for a number of circumstances, including standing (McLean and Goudy, 2004; Hodson-Tole et al., 2013; Reffad et al., 2014). Methodologically, these results suggest the local sampling of surface EMGs may not provide a representative view of the degree and duration of calf muscles' activation. Physiologically, the differential distribution of activity within the calf muscles may indicate a key mechanism contributing to the control of muscle force and thus of the standing posture. It is therefore possible that differences in the activation of postural muscles with aging are more expressive than previously appreciated.

This study questions, for the first time, whether the distribution of muscle activity differs between aged and young subjects during standing. Differently from previous studies, here we use arrays of surface electrodes to sample EMGs from different regions of individual, ankle muscles. More specifically, from surface EMGs collected serially from ankle plantar and dorsal flexors we ask: do young and aged subjects activate an equal proportion of their muscles for a similar duration during standing? If aging is associated with greater muscular effort for standing control (Nagai et al., 2011; Baudry et al., 2012), we therefore expect to observe EMGs with greater amplitude for a longer duration and in a larger muscle region in aged than young individuals.

## Materials and methods

### Participants

Thirteen young male volunteers provided written informed consent before participating in the study (mean ± SD; age: 26 ± 3 years; body mass: 72.4 ± 10.1 kg; height: 1.75 ± 0.06 m) and 11 aged (70 ± 6 years; 72.9 ± 12.5 kg; 1.72 ± 0.08 m). All participants were classified as minimally active according to the international physical activity questionnaire (IPAQ); short self-administered version (Tomioka et al., 2011). All community-dwelling older adults lived independently. We decided to include participants without a sedentary lifestyle because physical inactivity may further broaden the between-subjects variability often reported for stabilometric descriptors (Chiari et al., 2002). The experimental procedures considered in this study conformed with the *Declaration of Helsinki* and were approved by the Regional Ethics Committee (Commissione di Vigilanza, Servizio Sanitario Nazionale—Regione Piemonte—ASL 1—Torino, Italy). Volunteers did not report any balance impairments, neurological disorders, muscular injuries, or the intake of medications that could affect their standing balance at the occasion of experiments.

### Experimental protocol

**Figure 1:**
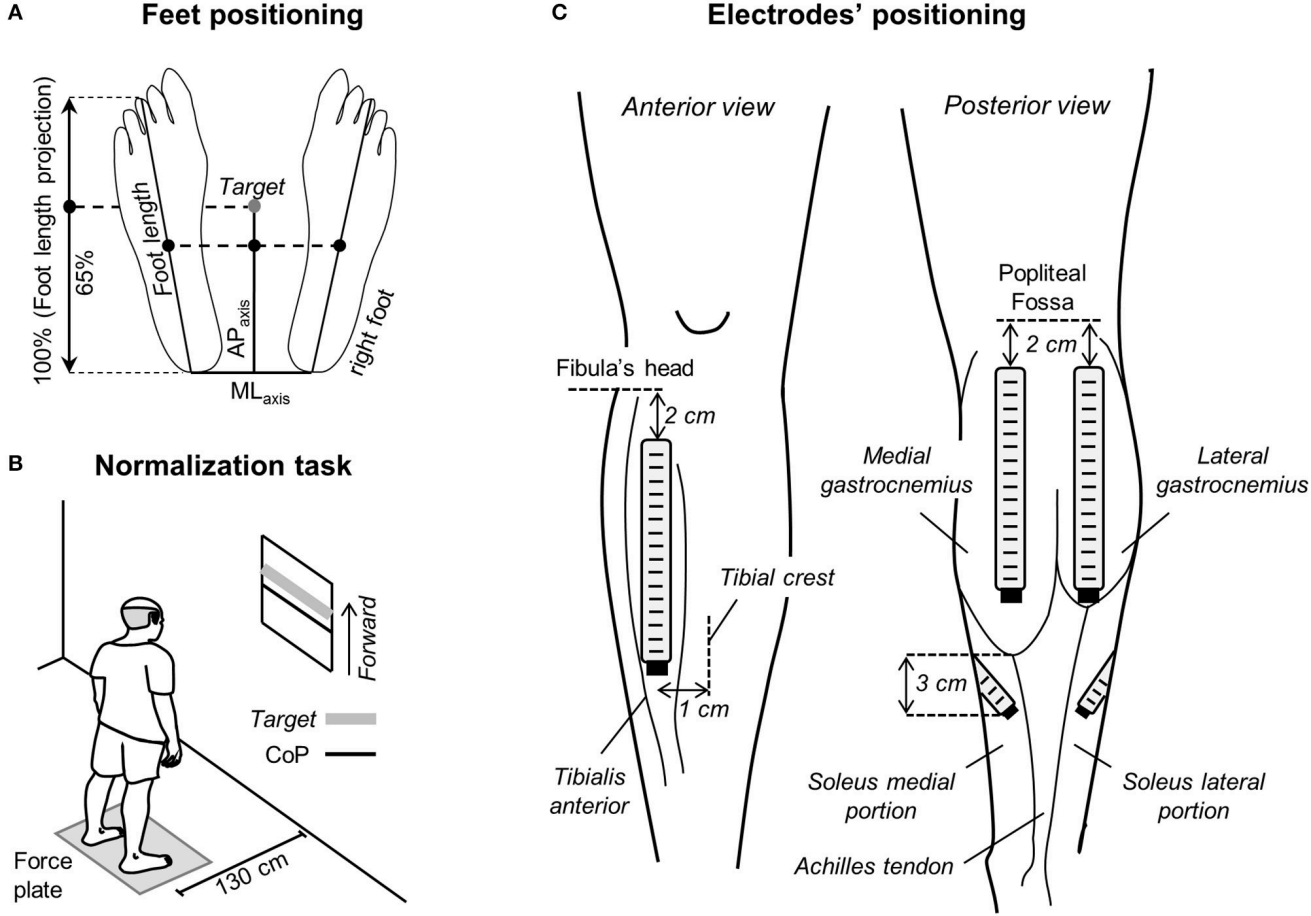
**Standing protocol and feet and electrode positioning. (A)** Schematic illustration showing the procedure considered to measure feet length. The midpoint between the tip of left and right calcaneus bones was considered as the origin of the reference system for center of pressure (CoP) and foot length measurements. The target value, corresponding to 65% of the foot length projected on the anterior-posterior axis (AP_axis_), was calculated and then considered for the normalization task shown in **(B)**. In such task, participants were asked to keep their CoP position in the AP_axis_ (black line) within 10% (±5%) of the target value (thick, gray line). **(C)** The positioning of electrode arrays is shown for the tibialis anterior muscle (left), for the medial and lateral gastrocnemius heads and for the soleus medial and lateral portions (right).

Participants were instructed to stand upright barefoot on a force-plate, with eyes open and arms alongside the body. They positioned their feet at a comfortable orientation and distance, while keeping heels and toes at the same position along the force plate anterior-posterior axis (Figure 1A). Prior to starting experiments, the contour of both feet was marked on the force plate to ensure participants would keep the same feet position throughout the standing tests.

Two standing tasks were applied. In the first task, participants were provided with visual feedback of their CoP position in the anterior-posterior axis and were instructed to keep it at 65% of the longitudinal size of their support base for 60 s (Figure 1B). The size of the support base in the anterior-posterior axis was defined as the distance between the tip of the third metatarsal head and the tip of the calcaneus bone projected in the anterior-posterior direction (Figure 1A). The 65% figure corresponds roughly to 80% of the distance between the heels and the anterior limit of stability of healthy, young subjects (Duarte and Zatsiorsky, 2002); this figure was selected to ensure a somewhat high degree of calf muscles active loading while not threatening stability, in particular for the aged individuals. All elderly subjects tested could stand with their CoP at the target value without losing balance. This postural task was considered for the normalization of EMGs, as described below. In the second task, volunteers were asked to stand at ease for 60 s. Subjects were engaged in active conversation to ensure they would take their mind off the test and thus avoid any voluntary change in muscle activity (Loram and Lakie, 2002b). The trial started over in the case gross body movements were noticed by the experimenter. The standing at ease task was applied three times, in accordance with previous evidence on excellent reliability of stabilometric descriptors (Pinsault and Vuillerme, 2009). Similarly, good-to-excellent reliability has been recently reported for EMGs detected from the calf muscles in aged individuals during standing (Gallina et al., 2016). Five minute intervals were applied between trials and their order was randomized.

### Electrodes' positioning

Linear arrays of surface electrodes were used to sample the distribution of ankle muscles' activity. Arrays were positioned over the plantar and dorsal flexors of both legs. Two arrays of 16 electrodes (10 mm inter-electrode distance) were used to detect surface EMGs from the medial and lateral gastrocnemius muscles. The most proximal electrode was located 2 cm distally to the popliteal fossa and arrays were aligned parallel to the longitudinal axis of each gastrocnemius' head (Figure 1C). Such positioning maximizes the representation of action potentials from muscle units residing in different, proximo-distal gastrocnemius' regions (Vieira et al., 2011). EMGs were sampled with one array of 16 electrodes (10 mm inter-electrode distance) from the tibialis anterior muscle, aligned 1 cm laterally and parallel to the tibial crest and with the most proximal electrode located 2 cm distally to the fibula's head (Figure 1C). Given the in-depth pennate architecture of tibialis anterior, as for gastrocnemius, such positioning is expected to provide EMGs representative of different, proximo-distal fibers. Two arrays with four electrodes each (10 mm inter-electrode distance) were used to sample EMGs from the soleus medial and lateral portions. For each soleus portion, arrays were aligned ~45 degree outward to the line connecting the junction between gastrocnemius' heads and the calcaneus tip. The lower border of both the medial and lateral arrays was positioned 3 cm distally to the medial gastrocnemius myotendinous junction (Figure 1C; Reffad et al., 2014). Gastrocnemii junction and their myotendinous junction were identified with ultrasound imaging (cf. Supplementary Material in Vieira et al., 2010b). Arrays were positioned after cleaning the skin with abrasive paste.

### Electromyographic and stabilometric recordings

EMGs were recorded in single-differential derivation. All 51 single-differential EMGs were amplified by a between-individuals variable factor—from 5,000 to 10,000—to ensure the highest signal-to-noise ratio without saturation (10–750 Hz bandwidth amplifier; EMG-USB, OTBioelettronica and LISiN, Politecnico di Torino, Turin, Italy). CoP coordinates in the sagittal and frontal planes were computed from the ground reaction forces supplied by a piezoelectric force-plate (9286AA Kistler, Zurich, Switzerland). Reaction forces and surface EMGs were sampled synchronously at 2,048 Hz using a 12-bit A/D converter (±2.5 V input dynamic range).

### Quantifying muscle activity during standing

Raw surface EMGs were visually inspected. Whenever any channel in the array presented contact problems, likely due to high skin-electrode impedance, or massive power line interference, the corresponding channel was disregarded. Occurrences of low quality EMGs were infrequent (17 out of 1,224 EMGs detected in total) and were observed mainly in one channel per array. After controlling for signal quality, EMGs from tibialis anterior and gastrocnemius muscles were inspected for the identification of propagating potentials. Propagating potentials may be observed in the distal muscle region, where surface electrodes and muscle fibers may run in parallel direction. In such case, different electrodes sample from the same group of fibers rather than from different, proximo-distal fibers (cf. Figure 1 in Hodson-Tole et al., 2013). Channels providing propagating potentials were therefore excluded from analysis; from 0 to 8 channels per array were excluded for the 24 participants tested.

After visual inspection, EMGs were band-pass filtered with a fourth order Butterworth filter (15–350 Hz cutoff; zero lag, bidirectional filter). Then, the Root Mean Square (RMS) was computed over 40 ms epochs (Laughton et al., 2003), providing a total of 1,500 RMS values per channel. From these RMS values, three indices were considered to assess for how long, how diffusely and how much elderly and young activated their ankle muscles during standing.

**Figure 2:**
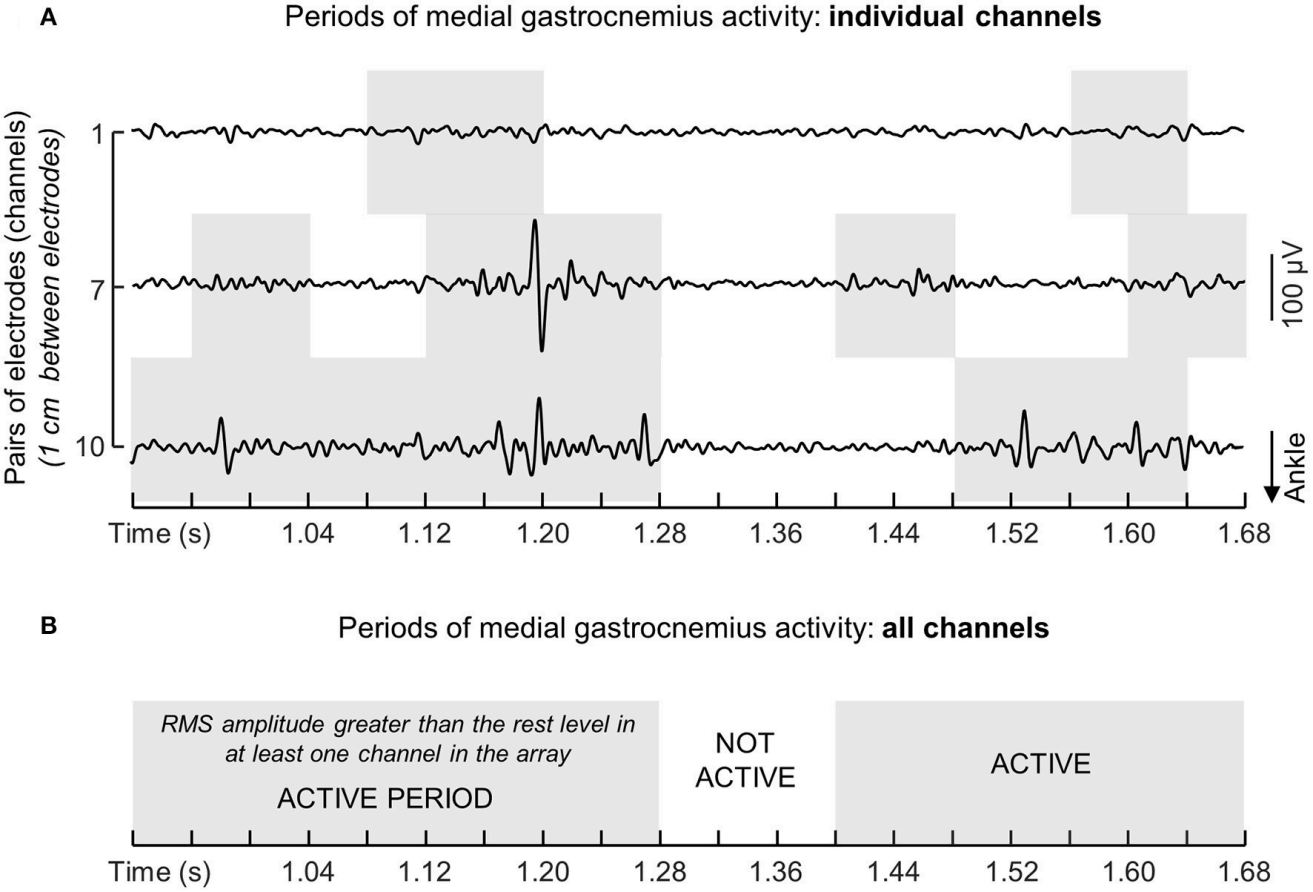
**Computation of instants of muscle activation. (A)** Shows examples of single-differential EMGs detected by channels 1, 7, and 10 from the medial gastrocnemius muscle of a single participant. Note action potentials do not appear with equally high amplitude in the three channels. The muscle was considered active whenever for any given instants the RMS amplitude exceeded the background activity in at least one channel. Light gray rectangles in the grid denote periods within which the RMS amplitude of EMGs in each channel exceeded the background activity computed with the muscle at rest (see text). The resulting series of instants of muscle activity for this representative example is illustrated with a succession of white (Inactive state; muscle at rest) and gray (Active state; muscle active) rectangles **(B)**.

Instants of activation were estimated by comparing the RMS values obtained during standing with the background activity. The background activity was defined from the RMS amplitude of EMGs collected with the ankle muscles at rest (Laughton et al., 2003). More specifically, for each channel in each array of electrodes we: (i) computed the RMS values over 40 ms epochs for EMGs detected during 3 s while participants were in supine position, providing a total of 75 RMS values; (ii) calculated the mean and the standard deviation for these RMS values; (iii) set the threshold defining the background noise level as the mean value plus three standard deviations; (iv) assigned Active or Inactive state to RMS samples respectively exceeding or not exceeding the background threshold. This procedure provided a series of Active-Inactive state values per channel. Given the EMGs detected by consecutive electrodes in the array sample from different fibers along the muscle proximo-distal axis, concurrent Active-Inactive events were often not observed between channels (Figure 2A). For this reason, to provide a global indication on the duration of muscle activity during standing, the muscle was deemed active whenever an Active state was observed across channels (cf. shaded areas in Figures 2A,B). The global series of Active-Inactive states indicates how long the ankle muscles were active throughout standing, regardless of where activity was observed in the muscle.

Based on the instants of activation, the spatial distribution and the intensity of muscle activity were computed. First, the number of channels detecting surface EMGs with RMS amplitude greater than 70% of the highest RMS amplitude in the array was identified. The 70% amplitude threshold was selected because it has been shown to provide a robust identification of channels located over active fibers within muscles pennate in the depth direction (Vieira et al., 2010a). The number of segmented channels multiplied by the inter-electrode distance was then normalized with respect to the muscle length (see below). Second, the degree of muscle activity was estimated by averaging the RMS values across all segmented channels for each muscle tested. This average RMS amplitude was then normalized with respect to the RMS amplitude averaged across channels identified during the normalization, standing task (Figure 1B), to compensate for the effect of anatomical differences between participants on the surface EMGs (Farina et al., 2002). It should be noted here the normalization of EMGs collected in a given condition with respect to that collected in a reference condition compensates for the effect of inter-individual differences on their amplitude though not on the spatial distribution of their amplitude. Finally, the size of the active region in the proximo-distal direction and the degree of activity were considered for analysis whenever any given muscle was active for at least 10% of the total, standing duration. Following previous evidence, occurrences of such sporadic activity was regarded as of marginal relevance for the control of standing posture (Héroux et al., 2014). Given we observed medial gastrocnemius was active for different durations (~20%) between legs in both aged and young subjects, the electromyographic indices and architectural muscle parameters (see below) computed from the subject's leg active for longer durations (left side for 10 young and 8 elderly subjects) were used for comparisons between groups.

### Measurements of ankle muscles' length and subcutaneous thickness

Parasagittal images from tibialis anterior and gastrocnemii were taken with a linear, ultrasound probe (10 MHz, 4 cm length; Echo Blaster 128, Telemed Ltd., Vilnius, Lithuania), with participants lying comfortably on a padded bed. Initially, the myotendinous junction was identified and marked on the skin. The length of tibialis anterior was then quantified as the distance between its myotendinous junction and the head of the fibula, whereas the length of each gastrocnemius head was defined as the shortest distance between the myoetendinous junction and the popliteal fossa. Subcutaneous thickness and pennation angle were further quantified to assist in the interpretation of potential proximo-distal differences in the distribution of activity with aging. Subcutaneous thickness was computed as the distance between the skin/fat layer over the muscle and its superficial aponeurosis from parasagittal images obtained with the probe centered halfway the muscle length. Thickness measurements were taken from the central region of the proximal, central and distal thirds of the ultrasound images and then averaged, providing a representative indication on the general subcutaneous thickness per subject (Onambele et al., 2006). Finally, the pennation angle was estimated as the angle between a clearly visible fascicle in the image and the muscle deep aponeurosis (Avancini et al., 2015). Thickness and pennation angle values were obtained with the muscle at rest.

### Quantifying the CoP sway area

The CoP sway area was considered to assess how largely young and elderly individuals swayed during the whole standing tests. The overall size of postural sways was estimated from the elliptic area conveying almost 85% of the total CoP samples during standing (Oliveira et al., 1996). The CoP elliptic area, as well as the EMG descriptors, were averaged across the three standing trials and considered for between-group comparisons. CoP data was 50 Hz low-pass filtered with a second order Butterworth filter to remove high-frequency noise.

### Statistical analysis

Non-parametric statistics were applied to compare the distribution of ankle muscles' activity between young and aged individuals, after ensuring the data distribution was not Gaussian (Shapiro-Wilk's *W*-test, *P* < 0.03 in all cases). The Mann-Whitney *U*-test was used to verify whether, during standing and for each muscle independently, the duration of the active period, the degree of activity, the relative size of the active region and the CoP elliptic area were different between groups. The same statistics was applied to assess regional differences in activity within soleus, by comparing the normalized RMS amplitude obtained for the muscle medial and lateral aspects. The level of statistical significance was set at 5% and data were reported using non-parametric, descriptive statistics.

## Results

### Representative examples of variations in ankle muscles' activity during standing

**Figure 3:**
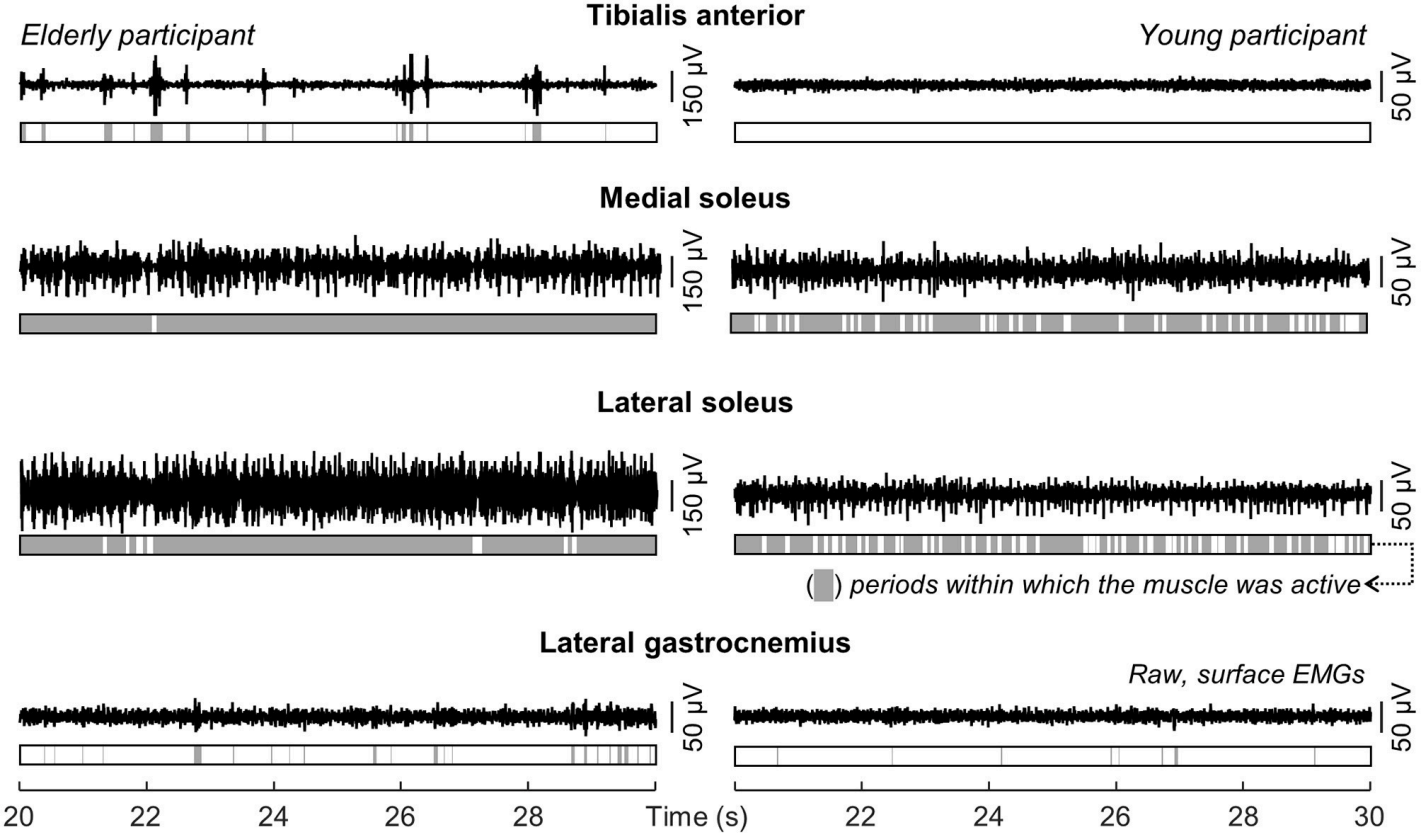
**Modulations in ankle muscles' activity during standing**. Single-differential EMGs collected for a representative channel from the tibialis anterior, the soleus medial and lateral portions and from the lateral gastrocnemius of a representative young and elderly participant are shown for 10 s of standing at ease. Light gray areas in the bars indicate periods within which the RMS amplitude exceeded the background activity (i.e., Active state).

Different ankle muscles were activated differently when young and aged individuals stood at ease. Descriptive considerations on these differences are summarized in this section based on the data shown in Figure 3 for one young and one aged, representative participant. Lateral gastrocnemius was active for 6 and 17% of the whole standing trial for the young and aged subjects respectively. For the tibialis anterior muscle, the amplitude of EMGs remained below the background activity for the young subject during the whole standing duration while, for the elderly, bursts of activity were observed and provided an active duration of 36%. Conversely, EMGs with remarkably high amplitude in the soleus medial and lateral portions were observed consistently for both subjects (cf. shaded areas in Figure 3).

**Figure 4:**
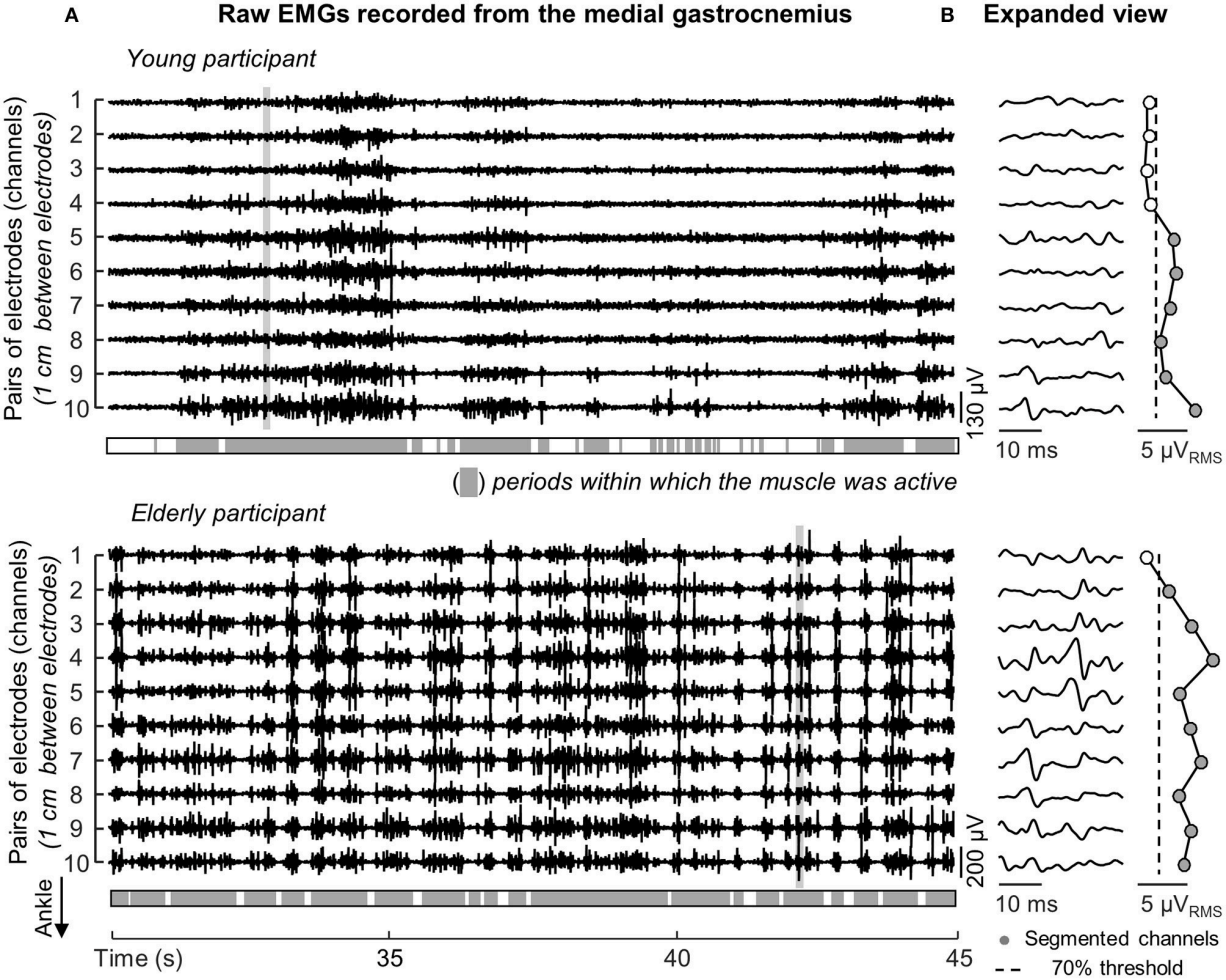
**Duration and distribution of medial gastrocnemius activity during standing**. Raw EMGs detected by channels located over the gastrocnemius superficial aponeurosis, from channel 1 to 10, are shown in **(A)** for a young and elderly representative participant. Light gray areas indicate periods within which the RMS amplitude exceeded the background activity. **(B)** Shows an expanded view of all EMGs. Note different action potentials appear in different channels for each subject. Note also there is no delay between potentials detected by consecutive channels (e.g., channels 1–6 for the aged participant). The distribution of RMS amplitude across channels is represented from circles, with gray circles indicating the *segmented channels*; that is, channels detecting largest EMGs in the array (see text).

The medial gastrocnemius showed a somewhat different pattern of activity when compared with the other ankle muscles. Differently from lateral gastrocnemius, soleus and tibialis anterior, medial gastrocnemius was not completely silenced, was not activated almost continuously and did not show sporadic bursts of activity. For both participants whose data is shown in Figure 3, alternate periods of medial gastrocnemius activation and silencing were observed consistently during standing (Figure 4A). When considering the distribution of activity within medial gastrocnemius, EMGs with relatively high amplitude were detected by six channels in the young and by nine channels in the aged subject (cf. gray circles in Figure 4B).

### Ankle muscles' activation in elderly and young individuals during standing

**Figure 5:**
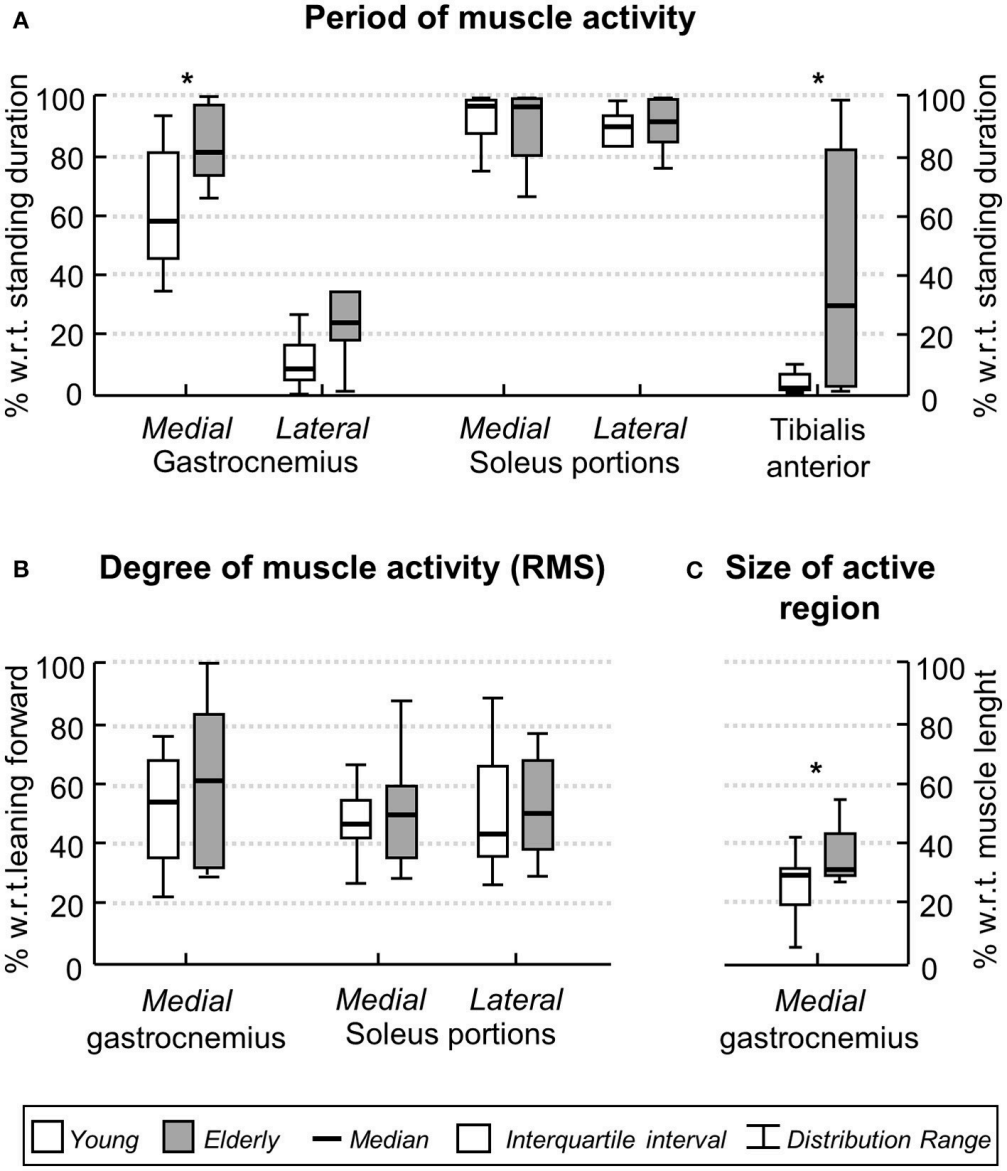
**Age-related differences in the ankle muscles' activity during standing**. Boxplots in **(A)** show the relative period within which, during standing, young (white boxes) and elderly (gray boxes) individuals activated their ankle muscles. Group data in **(B)** corresponds to the normalized, RMS amplitude obtained for both groups from the medial gastrocnemius and from the medial and lateral soleus portions. The proximo-distal size of medial gastrocnemius region over which largest EMGs were detected in both groups is shown in **(C)**. Asterisks indicate significant differences between groups (*P* < 0.05).

When considering group data, differences in the duration of ankle muscles' activity were observed between elderly and young subjects. The Mann-Whitney *U*-test revealed the medial gastrocnemius was activated for longer periods in aged (median, interquartile interval; 81.2, 74.1–98.2%) than young (58.8, 44.9–81.9%) individuals (Figure 5A; *P* = 0.02; *N* = 24; 13 young × 11 aged subjects). Similarly, notwithstanding the marked variability in the duration of active periods for the elderly, aged participants activated their tibialis anterior muscle during standing for a significantly longer duration than young subjects (*P* = 0.01; *N* = 24); tibialis anterior was rarely active in young (1.3, 0.7–4.4%) though not in the elderly (29.1, 2.6–82.5%). No group differences were observed in the duration of lateral gastrocnemius (*P* = 0.07) and soleus medial (*P* = 0.90) and lateral (*P* = 0.26) portions.

The degree and the distribution of activity within ankle plantar flexors varied differently between groups. The RMS amplitude of EMGs collected from the medial gastrocnemius and from the medial and lateral soleus portions did not differ between elderly and young (Figure 5B; *P* > 0.40 in all cases; *N* = 24). No differences were observed between the RMS amplitude for EMGs collected medially and laterally from soleus muscle, both for young and elderly (*P* > 0.70 in both cases). In contrast, for medial gastrocnemius, EMGs with relatively higher amplitude were detected over a significantly wider proximo-distal region in aged (32.5, 29.4–45.6%) than young (29.8, 20.1–31.3%) subjects (Figure 5C; *P* = 0.04; *N* = 24). Given the lateral gastrocnemius and tibialis anterior of young individuals were active for a somewhat short period during the whole standing tests (less than 10% on average; Figure 5A), these muscles were disregarded from further consideration.

### Medial gastrocnemius' subcutaneous thickness and pennation angle

No differences in subcutaneous thickness were observed between groups (*P* = 0.58). Thickness values ranged from 1.6 to 2.7 mm for young and from 1.6 to 3.5 for aged participants. Similarly, the pennation angle did not differ significantly (*P* = 0.37) between young (range: 19.6–24.0 degrees) and aged subjects (17.2–25.0 degrees).

### Differences in CoP sway area with age

While aged participants stood at ease, their CoP occupied an area roughly twice (median, interquartile interval; 4.4, 3.8–9.6 cm^2^) as large as that (2.3, 1.6–4.5 cm^2^) confining the CoP of young participants (*P* = 0.052).

## Discussion

In this study we used arrays of electrodes to investigate whether the temporal and spatial distributions of ankle muscles' activity differ between elderly and young individuals during standing. We hypothesized that elderlies would present greater EMGs, distributed over a larger muscle region and for a longer duration than young individuals. Surface EMGs from different regions of the ankle muscles were collected while subjects stood at ease to test this hypothesis. Our key results indicate that during standing: (i) tibialis anterior and medial gastrocnemius muscles were active for a longer duration in aged than young subjects; (ii) a greater proportion of medial gastrocnemius volume was active in elderlies. Collectively, these results indicate elderlies rely more heavily on the active loading of ankle muscles to control their standing posture than young individuals.

### Preliminary, methodological considerations on surface EMG detection

Differently from previous studies, here we sampled surface EMGs with arrays of electrodes (Figure 1C). Our decision to sample activity from multiple locations of individual ankle muscles was motivated by recent evidence suggesting different muscle regions may be activated differently (Brown et al., 2007; Farina et al., 2008), in particular for pennate muscles (McLean and Goudy, 2004; Staudenmann et al., 2009; Vieira et al., 2011; Hodson-Tole et al., 2013); EMGs sampled locally (i.e., with a single pair of shortly spaced electrodes) may not provide a representative view of muscle activation. Indeed, as shown in Figure 2, EMGs in different locations may provide different estimations for the duration of gastrocnemius activity. Considering EMGs were represented with different amplitude in different muscle regions (Figures 2, 4), the duration of muscle activity would have been likely underestimated if we had not considered EMGs detected at different skin regions (cf. shaded areas for individual and all channels in Figure 2). Similarly, biased estimations on the degree of each calf muscles' activity would have been obtained if we had considered the RMS amplitude of EMGs detected locally. Depending on where EMGs were detected from, in particular for young individuals (Figure 4), their corresponding RMS values would provide either under- (white circles in Figure 4B) or over- (gray circles in Figure 4B) estimates of the degree of activity in the whole muscle. Through arrays of surface electrodes, we were able to obtain estimates of the duration and degree of activity presumably more representative of individual ankle muscles' volumes than previously appreciated. As discussed below, new insights have been gained into age-related differences in postural activation from such a high-density, surface EMG approach.

### Are the ankle muscles activated for a similar duration in aged and young subjects?

The differences between aged and young individuals reported here were muscle dependent. When considering the duration of activity for lateral gastrocnemius and soleus muscles, statistic differences were not observed between groups. Both groups recruited lateral gastrocnemius for less than ~20% of the total, standing duration (Figures 3, 5A). The soleus muscle, on the other hand, was activated for periods roughly longer than 80% of standing. These results corroborate previous findings on the duration of calf muscles' activity, extensively reported for young subjects. Indeed, while the absence of activity in lateral gastrocnemius has been observed in some subjects (Masani et al., 2013; Héroux et al., 2014), it seems well-documented that soleus is recruited at almost all time during standing (Laughton et al., 2003; Héroux et al., 2014). Even though the temporal activation of postural muscles is not as commonly assessed for elderlies as it is for young individuals, Laughton et al. (2003) observed age similarities for the duration of soleus activity during standing. Extending the observation of Laughton et al. (2003), our current results suggest the duration of activity of the lateral gastrocnemius and soleus regions accessed by our surface electrodes is unlikely sensitive to aging.

Age differences emerged however in the duration of medial gastrocnemius and tibialis anterior activity during standing. Aged individuals activated their medial gastrocnemius muscle for a period ~20% longer, on average, than their young counterparts (Figure 5A). Similarly, tibialis anterior was active for a longer (~30%) period in aged than in young subjects (Figure 5A). The median duration of tibialis anterior activity observed here, both for young (~2%) and elderlies (~30%), is well in agreement with the duration of activity periods reported by Laughton et al. (2003) for this muscle. From a first inspection of results presented in Figure 5A, we feel inclined to consider the co-activation mechanism as responsible for the age differences in the timing of plantar and dorsal flexors' activation during standing. More specifically, while young subjects predominantly activated their plantar flexors, elderlies activated both plantar and dorsal flexor muscles during standing (Figures 3, 4A, 5A). In spite of controversies on whether co-activation may be detrimental or may compensate for poor control of posture, co-activation is often associated with increased postural sways (Nagai et al., 2011; Baudry et al., 2012; Warnica et al., 2014). Corroborating this common view, in the elderlies, we observed the CoP was confined within an elliptic area almost twice as large as that confining the CoP of young subjects. Regardless of the mechanisms underpinning age-differences in posture control, current results here suggest elderlies activate their ankle muscles for longer durations during standing than young subjects.

### Do elderly and young recruit their calf muscles to a similar extent during standing?

Two notes on our estimates of the degree of activity are necessary before interpreting results. Young subjects activated tibialis anterior and lateral gastrocnemius somewhat rarely (Figures 3, 5A). We therefore disregarded both muscles from age comparisons. A second observation concerns how we conceived muscle activity. While the amplitude of EMGs is traditionally considered to assess the degree of muscle activity, here we considered both the amplitude and the size of skin region where EMGs with relatively high amplitude were sampled (Figures 2, 4). As argued below, the extent to which elderly and young activate their calf muscles depends both on the amplitude and on the amplitude distribution of surface EMGs.

The proximo-distal distribution of the amplitude of medial gastrocnemius EMGs distinguished aged from young individuals during standing. While young subjects presented EMGs with relatively high amplitude in the gastrocnemius distal region, corroborating previous findings (Hodson-Tole et al., 2013), EMGs with similarly high amplitude were sampled from a larger, proximo-distal muscle region in aged individuals (c.f. gray circles in Figures 4B, 5C). Different sources could have accounted for a somewhat extensive distribution of EMG amplitude in the elderlies. A first issue to consider is the potential difference in muscle architecture between groups. Both subcutaneous thickness and pennation angle have shown to affect dramatically the amplitude distribution of surface EMGs; thicker subcutaneous tissue and smaller pennation angle result both in more spatially diffused surface EMGs (Farina et al., 2002; Mesin et al., 2011). Current results however did not indicate significant, age differences in gastrocnemius architecture. The lack of anatomical differences between groups leads us to consider the possibility that elderlies activated a larger gastrocnemius volume than young subjects during standing. In spite of recent controversies (Blouin et al., 2016; Vieira et al., 2016), it seems well-accepted that, for the gastrocnemius muscle, EMGs sampled in different proximo-distal regions reflect the activity of different muscle fibers (Vieira et al., 2011). The amplitude of EMGs detected distally and proximally, for example, is associated with the number of active fibers in the muscle distal and proximal regions respectively (Mesin et al., 2011). Given the mean amplitude of EMGs sampled from medial gastrocnemius was similar between groups (Figure 5B), the wider skin region from where EMGs with high amplitude were detected (Figure 5C) suggests a relatively greater proportion of muscle fibers may have been recruited in the elderlies.

Age differences in the distribution of activity for soleus were not as clear as for gastrocnemius. Since soleus is largely covered by the gastrocnemius muscles, spatial differences in soleus activation were assessed by comparing the amplitude of EMGs detected laterally and medially (Figure 1C). Our decision to assess both regions was based on previous evidence showing medio-lateral differences in soleus activation (Staudenmann et al., 2009; Reffad et al., 2014). Given we were here interested in assessing the degree of muscle activity during standing, sampling EMGs unilaterally could provide a biased view on the actual degree of soleus, postural activity. Indeed, even though results in Figure 5B indicate EMGs with equal amplitude were detected medio-laterally for both age groups, stating young and aged subjects activate a similar proportion of their soleus muscle in standing is potentially fairly speculative. Considerations on the relevance of spatial differences in soleus activation with age are therefore conditioned to the possibility of sampling EMGs from a greater soleus region (with e.g., intramuscular electrodes) in future investigations.

### Future perspectives and limitations

Spatial differences in the amplitude of EMGs collected from elderlies and young during standing have methodological and physiological implications. A first crucial point to consider is the localized representation of surface EMG during standing. Here we show that, depending on where a single bipolar EMG is collected from the gastrocnemius muscle, different conclusions maybe drawn on age differences during standing. The local sampling of muscle activity provided by bipolar surface EMGs may indeed contribute to explaining current disparities observed in the literature. While some studies did not report age differences in the degree of plantar flexors' activity during standing at ease (Amiridis et al., 2003; Laughton et al., 2003), others have however documented a significantly higher degree of plantar flexor activation with aging (Nagai et al., 2011; Baudry et al., 2012). Anticipating the differences between studies were due to inappropriate EMG sampling is a statement we strongly discourage. On the other hand, though, our current results suggest that different interpretations may emerge from EMGs detected locally during standing. Of more physiological, applied interest, is the suggestion that elderlies tend to stand with a greater degree of muscle effort than young subjects (Figures 5A,C). According to recent evidence, standing with minimal muscular effort while affording some degree of bodily sways may be advantageous; it may reduce the metabolic cost of standing (Bottaro et al., 2008), eliminate motor noise (Loram et al., 2011) and compensate for delays in the feedback loop (Cabrera and Milton, 2002). While acknowledging the value of different protocols so far devised for the balance training (Li et al., 2005; Sayenko et al., 2010), here we suggest that learning to efficiently activate dorsal and plantar flexors during standing could be beneficial for the control of standing posture in aged individuals.

Some additional considerations on the results presented here are necessary. Both young and aged subjects showed a somewhat large variability in the duration of ankle muscles activity (Figure 5A). A first possible explanation to such inter-individual variability is the difference in ankle stiffness between subjects (Loram and Lakie, 2002a). Additionally, the inter-individual variability in muscle activation during standing, in particular the duration of tibialis anterior activation in the elderly, may be due to different neural sources. With aging, an assortment of impairments arising at the peripheral and central levels of the nervous system may develop, each impacting detrimentally and differently on the control of the standing posture (Horak et al., 1997). By testing subjects without a sedentary lifestyle we expect to have limited the repertoire of posture-related impairments affecting present results. A final consideration regards whether crosstalk could have affected our results. We believe this possibility is unlikely. First because action potentials were represented locally in the surface EMGs. For example, the fact that action potentials detected from a given gastrocnemius region did not appear in neighbor channels (cf. Figure 2 and expanded view of EMGs in Figure 4) suggest any crosstalk from muscles other than gastrocnemius was relatively marginal. Moreover, the duration of activity of both muscles would be the same if crosstalk from soleus had contributed substantially to EMGs collected from gastrocnemius. In spite of these considerations, our results show the duration and the region of muscle activity, as quantified from surface EMGs, discriminate well-aged from young individuals during standing.

## Author contributions

FD and TV were involved with all aspects of the study. TP contributed to acquire, analyse and interpret the data. MG contributed to analyse and interpret the data. All authors contributed to draft the work and revise it critically.

## Funding

This study was supported by a national research project funded by the Italian Ministry of Education, Universities and Research (Protocol number: 2010R277FT), and cofunded by Compagnia di San Paolo and Fondazione C.R.T. FD is recipient of a scholarship provided by Coordenação de Aperfeiçoamento de Pessoal de Nível Superior / Ciência sem Fronteiras / Processo n° BEX 9404/13-9. TPP is recipient of a scholarship provided by Coordenação de Aperfeiçoamento de Pessoal de Nível Superior / Ciência sem Fronteiras / Processo n° BEX 9130/13-3. The funders had no role in study design, data collection and analysis, decision to publish, or preparation of the manuscript.

### Conflict of interest statement

The authors declare that the research was conducted in the absence of any commercial or financial relationships that could be construed as a potential conflict of interest.
